# Quality of Life Is Associated With Wearable-Based Physical Activity in Patients With Inflammatory Bowel Disease: A Prospective, Observational Study

**DOI:** 10.14309/ctg.0000000000000094

**Published:** 2019-11-01

**Authors:** Miriam Wiestler, Fabian Kockelmann, Momme Kück, Arno Kerling, Uwe Tegtbur, Michael P. Manns, Masoumeh Attaran-Bandarabadi, Oliver Bachmann

**Affiliations:** 1 Department of Gastroenterology, Hepatology and Endocrinology, Hannover Medical School, Hannover, Germany;; 2 Institute of Sports Medicine, Hannover Medical School, Hannover, Germany;; 3 Current affiliation: Siloah St. Trudpert Klinikum, Pforzheim, Germany.

## Abstract

**OBJECTIVES:**

Patient-reported outcomes such as quality of life are gaining importance in the assessment of patients suffering from inflammatory bowel disease (IBD). The association of objectively measured physical activity and quality of life in patients with IBD has not been studied in depth. To investigate the association of disease-specific quality of life and physical activity as well as clinical and biochemical disease activity in patients with IBD.

**METHODS:**

A total of 91 patients with IBD were stratified into 4 groups (Crohn's disease and ulcerative colitis, in remission and with moderate-severe activity, respectively) and evaluated in terms of disease-specific quality of life (Inflammatory Bowel Disease Questionnaire [IBDQ]), physical activity (accelerometry), body composition (bioelectrical impedance analysis), as well as clinical (Harvey-Bradshaw Index and Simple Clinical Colitis Activity Index) and biochemical (C-reactive protein and fecal calprotectin) parameters of disease activity.

**RESULTS:**

In patients with moderate-severe disease activity, the IBDQ was significantly lower as compared to patients in remission (Mann-Whitney *U* test and Kruskal-Wallis test, *P* < 0.001). The physical activity level was higher in remission than in active disease (Mann-Whitney *U* test, *P* < 0.05). The IBDQ was significantly correlated with the duration of strenuous physical activity per day (*P* = 0.029178, r = 0.235), skeletal muscle mass (*P* = 0.033829, r = 0.229), and biomarkers of inflammation (C-reactive protein: *P* < 0.005, r = −0.335 and fecal calprotectin: *P* < 0.005, r = −0.385).

**DISCUSSION:**

In this prospective, cross-sectional study, disease-specific quality of life was significantly associated with accelerometrically determined physical activity and disease activity in patients with IBD. This may be related to a reciprocal impact of these factors (DRKS00011370).

## INTRODUCTION

Patients with inflammatory bowel disease (IBD) are at risk of an impaired quality of life (1,2). In a recent meta-analysis, quality of life was poorer during active disease and presumably poorer in patients with Crohn's disease (CD) (3). Health-related quality of life (HRQoL) of patients with CD is consistently determined by markers of disease activity, including work disability, evidence of intestinal inflammation, number of relapses, biological treatment, and hospitalization rate (4).

Early on, studies on the general population have investigated the impact of physical activity on quality of life. Data from the Health Survey for England show that high levels of activity are associated with better HRQoL and that this link is particularly found using objective measures as compared to surveys (5). The implications of physical activity in patients with IBD have been investigated with respect to both exercise programs and habitual physical activity. After early studies were intended to verify the safety of exercise in IBD (6), there is now good evidence that exercise programs are well tolerated in IBD, and some studies even reported a decrease in disease activity (reviewed in (7)).

Habitual physical activity, however, may represent an aspect which is more meaningful for everyday life in patients with IBD, and furthermore, not all patients are amenable to exercise programs (8). From the available literature, it can be concluded that the level of habitual physical activity found in patients with IBD is invariably lower than that of controls and below the current minimum recommendations for general health (7). In most studies, physical activity was quantified using surveys (9,10), which may be prone to reporting bias. Evidence from studies with objective measures of physical activity, such as wearable devices, is sparse and often focuses on a selected patient group, such as IBD in remission, pediatric patients, or CD alone (11,12).

The present prospective, cross-sectional study was devised to analyze habitual physical activity in adult CD and ulcerative colitis (UC) patients using accelerometry as an objective measure. Taking into account the range of different IBD activity states, patients were stratified into groups including patients with active and quiescent disease for each disease entity. The main objective of this study was to investigate the link between habitual physical activity and disease-specific quality of life in IBD.

## MATERIALS AND METHODS

### Study design

We performed a prospective, monocentric, cross-sectional, observational study. It complies with the Declaration of Helsinki (2013), was approved by the institutional ethics committee (No. 7275/2016), and was registered at the German Clinical Trials Register (DRKS) as DRKS00011370. Written informed consent was obtained from all patients. The manuscript was drafted in accordance with the STROBE (STRengthening the Reporting of OBservational studies in Epidemiology) checklist (13).

### Participants and setting

From January 2017 until October 2017, 91 patients with verified diagnosis of UC or CD and a disease duration of at least 3 months were recruited during their visits at the IBD outpatient clinic of Hannover Medical School. Inclusion criteria were patients older than 18 years, confirmed diagnosis of UC or CD, and disease activity within a defined range. Exclusion criteria were stoma, pregnancy, and limitations in everyday life caused by cardiovascular or orthopedic diseases. For each disease entity, patients were stratified into a “remission” (defined as a Harvey-Bradshaw Index [HBI] of <5 (14) or a Simple Clinical Colitis Activity Index [SCCAI] of <4 (15), respectively) or “active disease” (HBI > 7 or SCCAI > 6, respectively) group to obtain a wide spectrum of disease activities for later correlation analysis.

### Study aims and variables

Baseline variables included age, sex, smoking status, employment status, and previous anti-inflammatory therapies. Primary study aim was the correlation of quality of life with parameters of physical activity, body composition, as well as clinical and biochemical features of disease activity. Secondary study aim was comparison of the remission group and the active disease group in terms of quality of life, physical activity, body composition, disease activity, and laboratory values.

### Questionnaires

Questionnaires were completed by the recruiting physician and the patient. Survey items included HBI or SCCAI (depending on the disease entity), education and employment status, medical history, and specific information on actual and former medication, as well as surgical interventions. The International Physical Activity Questionnaire (IPAQ) was used to assess self-administrated physical activity during the preceding week (16) and the IBD Questionnaire to measure quality of life (17).

### Bioelectrical impedance analysis

Total body composition was measured using bioelectrical impedance analysis (InBody 720, Inbody, Cerritos, CA). To minimize interindividual differences, patients were asked to undress except for underwear and to urinate before starting the measurement. The electrodes of the device were accurately cleaned before every use. While the patients stood on feet electrodes and held on to hand electrodes, body composition was analyzed through 8 electrodes and multiple frequencies in 5 body segments.

### Physical activity measured by accelerometry

All patients were instructed to wear a biaxial accelerometer (BodyMedia, Pittsburgh, PA) with acceleration sensors for longitudinal and transversal movement, 2 temperature sensors, a heat flow sensor, and a galvanic skin sensor to analyze and measure duration and depth of physical activity. Together with personal information such as weight, height, age, and smoking status, accelerometric data were used to calculate parameters of physical activity with context-specific linear regression. Data analysis was performed using the manufacturer's software package (SenseWear Professional 6.1) readout including the duration of different levels of physical activity: Moderate activity was defined as activity between 3 and 6 metabolic equivalents (METs), vigorous activity between 6 and 9 METs, and very vigorous activity over 9 METs. In addition, the number of steps, average of METs, the physical activity level (PAL) per day, duration of recumbency, and duration of sleep were monitored. Sleep efficiency (duration of sleep/duration of recumbency) and the number of burnt calories (kcal) were calculated. To get an exact history of physical activity for each subject per day, the accelerometer initiated 1 measurement per minute.

At the beginning, participants were trained in the use of the accelerometer, which was fit to the right upper arm (middle of the humerus closely to the musculus triceps brachii) using an elastic belt. All participants were instructed to wear the accelerometer 24 hours a day, except for the time of showering and swimming because the accelerometer is not waterproof. To get a realistic profile of physical activity, all participants were told to wear the accelerometer continuously for 7 days after their outpatient visit.

### Laboratory values

Blood and fecal samples were collected during the participants' inclusion into the study. Biochemical parameters included hemoglobin (g/dL), leukocyte counts (1.000/μL), ferritin (μg/L), transferrin saturation (TSAT) (%), protein (g/L), C-reactive protein (CRP) (mg/L), and vitamin D3 (ng/mL). Calprotectin (mg/kg) was measured in fresh fecal samples. All laboratory tests were performed by using routine methodology in the laboratory of Hannover Medical School.

### Statistical methods

Data were analyzed using SPSS Statistics software version 25.0 (SPSS, IBM, Armonk, NY). Normal distribution was assessed by the Shapiro-Wilk test, where *P* > 0.05 shows normality of data. Most variables in our analysis did not show normal distribution, so we chose nonparametric statistics (e.g., median for data display, the Mann-Whitney *U* test to compare the “active disease” and “remission” groups). Correlation analysis was performed using Spearman's rank coefficient.

## RESULTS

**Figure 1.**
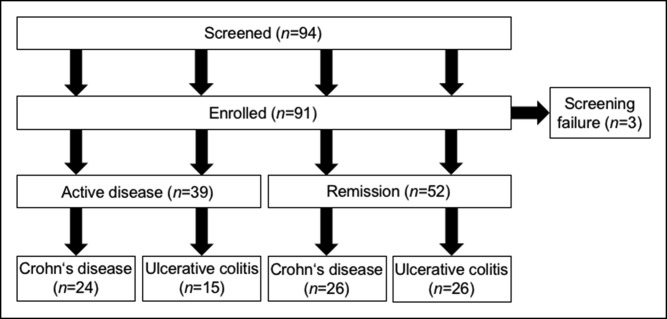
Patient disposition: According to the protocol, patients were stratified into 4 groups (Crohn's disease and ulcerative colitis patients with active disease and in remission, respectively). Three patients were screening failures due to stoma. N is given in brackets.

**Figure 2.**
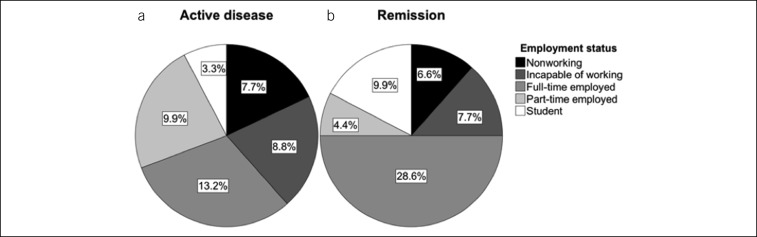
Employment status in patients with active disease (**a**) and in remission (**b**). No significant differences in employment status between the “active disease” vs “remission” groups were detected (n = 39 vs 52, chi-quadrat test of Pearson, *P* = 0.074).

A total of 94 subjects were screened, and 91 were enrolled into the study. In accordance with the predefined stratification criteria (see Materials and Methods), 39 patients had active disease (CD, n = 24 and UC, n = 15), and 52 were in remission (CD, n = 26 and UC, n = 26; Figure 1). Baseline characteristics were well equilibrated between the “active disease” and “remission” groups. In particular, no significant differences were noted for sex, age, smoking status, and previous anti-inflammatory therapies, the latter being an indirect marker of disease severity (Table 1). As the employment status may markedly influence habitual physical activity, questionnaires also inquired whether a patient was not working, incapable of working, full-time employed, part-time employed, or studying. Importantly, no significant differences were noted between the groups regarding employment status either (Figure 2).

**Table 1.**
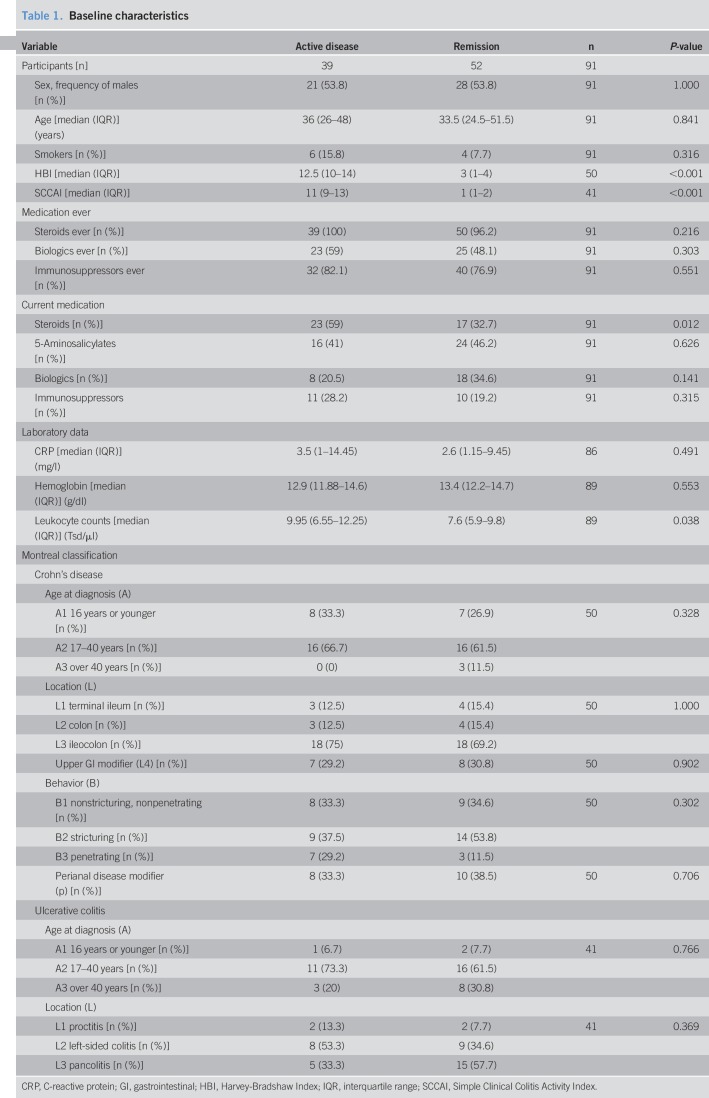
Baseline characteristics

**Figure 3.**
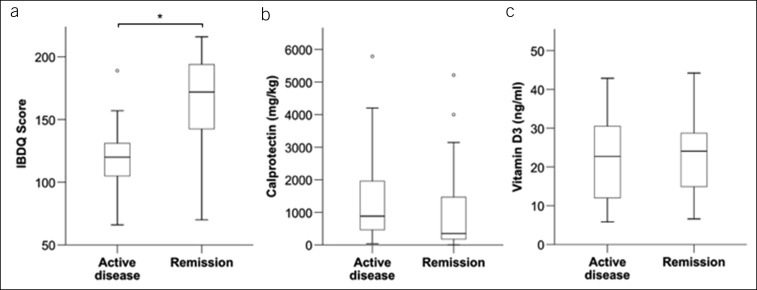
Health-related quality of life and biomarkers of inflammation in patients with active disease vs remission: (**a**) IBDQ score was significantly higher in patients in remission than with active disease (120 [105–132] vs 172 [141–194]; n = 86; *P* = 0.00000002; median [IQR]; Mann-Whitney *U* test). (**b**) Median fecal calprotectin was lower in patients in remission, but the difference between the groups was not significant (883.25 [436.65–2059.08] vs 355.2 [173.75–1,489.45] mg/kg; n = 58; *P* = 0.062; median [IQR]; Mann-Whitney *U* test; outlier value in the active disease group not shown [7,952.9]). (**c**) Vitamin D3 levels were not different between both groups (22.1 [10.65–30.05] vs 24.1 [14.55–29.25] ng/mL; n = 78; *P* = 0.332; median [IQR]; Mann-Whitney *U* test; 3 patients had values below the limit of detection [<0.3 ng/mL] and were excluded; *P < 0.05). IBDQ, Inflammatory Bowel Disease Questionnaire; IQR, interquartile range.

**Figure 4.**
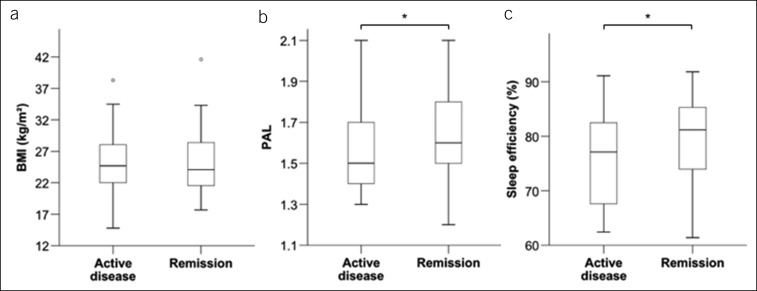
Body mass index, energy expenditure, and sleep efficiency in patients with active disease vs remission: (**a**) BMI was comparable in both groups (24.7 [21.6–28.1] vs 24.1 [21.53–28.5] kg/m^2^; n = 91; *P* = 0.773; median [IQR]; Mann-Whitney *U* test). (**b**) Physical activity level measured by accelerometry was significantly lower in patients with active disease (1.5 [1.4–1.7] vs 1.6 [1.5–1.8]; n = 91; *P* = 0.04979; median [IQR]; Mann-Whitney *U* test; active disease group: two outlier values not displayed [2.2; 2.7]; remission group: two outlier values not displayed [2.3; 2.4]). (**c**) Sleep efficiency was significantly lower in patients with active disease (77.1 [67.6–83] vs 81.15 [73.78–85.35] %; n = 91; *P* = 0.041; median [IQR]; Mann-Whitney *U* test; active disease group: four outliers not displayed [31.6; 38.5; 42.9; 43.1]; remission group: one outlier value not displayed [37.8]; *P < 0.05). BMI, body mass index; IQR, interquartile range; PAL, physical activity level.

Next, group comparisons were performed between the “active disease” and the “remission” groups (figure 3 and 4). The Inflammatory Bowel Disease Questionnaire (IBDQ) score was significantly higher in patients who were in remission of their IBD, a finding that is well supported by the published literature (1–4) (Figure 3a). In addition, a lower score was found in the “active disease” group for all 4 subcategories of the IBDQ (social, emotional, system, and bowel; supplementary data). Biochemical markers of disease activity/severity that were measured in the study included fecal calprotectin, which showed a nonsignificant trend toward higher levels in the “active disease” group, and vitamin D, which was not different (Figure 3b,c).

Eighty-six patients (35 in the “active disease” and 51 in the “remission” group) filled in the IPAQ. With the information of the questionnaire, we calculated minutes of vigorous, moderate, and sedentary activity per week and duration of activity by feet per week. Three patients (2 in the “active disease” and 1 in the “remission” group) indicated “no activity/I don't know” for the variables vigorous, moderate, and sedentary activity per week. There were 12 patients (7 in the active disease and 5 in the remission group) who indicated “I'm not sure/I don't know” for the variable “sedentary activity.” These data points were omitted from the analysis.

As an important aspect which may be affected by disease activity, and may in turn show reciprocal influence on habitual physical activity, parameters of body composition were analyzed. Body mass index (BMI) was not statistically different between the groups (Figure 4a).

A primary aim being the investigation of objectively measured habitual physical activity in IBD, accelerometric parameters were compared between the “active disease” and the “remission” group. There was a strong indication for decreased physical activity in the active disease group, shown by a significantly lower PAL (Figure 4b). Total steps per day were numerically lower in the “active disease” group than in the remission group, but this finding failed to reach statistical significance. In addition, duration of vigorous and very vigorous activity showed a trend toward higher levels in the “remission” group. Furthermore, sleep patterns were accelerometrically investigated. Although total lying and sleeping times per day were similar between the “active disease” and “remission” groups, patients with active disease had a significantly lower sleep efficiency (Figure 4c).

**Figure 5.**
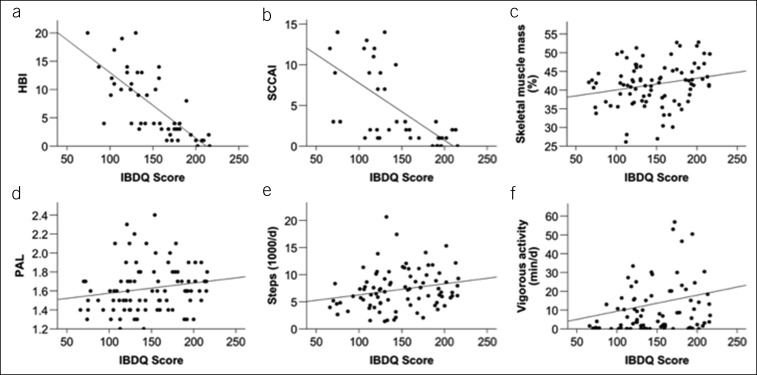
Correlation of HRQoL with disease activity, body composition, and physical activity: (**a** and **b**) There was a strong inverse correlation between IBDQ and IBD activity, shown for the Harvey-Bradshaw Index (r = −0.788; *P* < 0.001; n = 46) and Simple Clinical Colitis Activity Index (r = −0.685; *P* = 0.001; n = 40). (**c**) As an exemplary parameter from body impedance analysis, skeletal muscle mass correlated positively with the IBDQ score (r = 0.229; *P* = 0.034; n = 86). (**d**–**f**) Analysis of 1-week accelerometry data showed that physical activity level (PAL) as a measure of energy expenditure, steps per day, and vigorous activity all correlated positively with HRQoL (**d** [PAL]: r = 0.234; *P* = 0.030; **e** [steps per day]: r = 0.260; *P* = 0.016; and **f** [vigorous activity]: r = 0.235; *P* = 0.029; n = 86 in each analysis). HBI, Harvey-Bradshaw Index; HRQoL, health-related quality of life; IBD, inflammatory bowel disease; IBDQ, Inflammatory Bowel Disease Questionnaire; PAL, physical activity level; SCCAI, Simple Clinical Colitis Activity Index.

Next, we focused on quality of life, as previous studies had reported an association with disease activity (1–4) and also with physical activity in selected aspects (11,12). As expected, the IBDQ was significantly associated with the disease activity indices HBI and SCCAI, respectively (Figure 5a,b). In terms of laboratory values, there were associations with CRP, protein, and fecal calprotectin as surrogate markers of disease activity (see Table 1, Supplementary Digital Content 1, http://links.lww.com/CTG/A116).

We then asked whether quality of life is linked to parameters of body composition and habitual physical activity. Indeed, biometrical impedance analysis data significantly correlated with the IBDQ with respect to total body water, protein, fat, and total muscle mass (the latter shown in Figure 5c, further parameters in Table 1 [Supplementary Digital Content 1, http://links.lww.com/CTG/A116]). Importantly, parameters of physical activity were significantly correlated with the IBDQ. Correlation analysis of 1-week accelerometry data showed that the PAL as a measure of energy expenditure was significantly associated with quality of life (Figure 5d). In addition, steps per day (Figure 5e), vigorous activity (Figure 5f), and sleep efficiency (see Table 1, Supplementary Digital Content 1, http://links.lww.com/CTG/A116) were significantly associated with the IBDQ. From the IPAQ, calculated vigorous activity per day was associated with the IBDQ (see Table 1, Supplementary Digital Content 1, http://links.lww.com/CTG/A116).

## DISCUSSION

The aim of this study was to analyze the interrelationship between HRQoL, habitual PALs, and disease activity in patients with IBDs. First, our findings demonstrate a correlation between disease activity and quality of life in patients with IBD, which is consistent with already published data. Second, the results presented here indicate a significant correlation between habitual PALs and IBD activity, as evidenced by significantly higher PAL levels in remission vs active disease. Moreover, and importantly, these data positively correlate with HRQoL and further demonstrate the positive effect of physical activity for patients with IBD.

In the current study, we used an accelerometric approach to objectively measure the patients' habitual physical activity. The SenseWear Armband has been validated as a useful tool to closely estimate energy expenditure. In a Spanish study, 23 healthy adults aged between 40 and 55 years were asked to perform standardized physical activities while measuring their energy expenditure using the SenseWear Armband and through indirect calorimetry (18). The authors were able to demonstrate that the SenseWear Armband overestimated energy expenditures for all activities (rest, walking at 3 and 5 km/hr, running at 7 and 9 km/hr, and sitting/standing with a chair) compared with indirect calorimetry. On the other hand, Drenowatz and Eisenmann (19) concluded from their study that high-energy expenditure measurements with the SenseWear Armband show a ceiling effect around an intensity of 10 METs. In this study on 20 endurance-trained subjects with a median age of 24.3 (±2.8) years, probands were asked to perform well-defined physical activities (three 10-minute treadmill runs at 65%, 75%, and 85% of their VO_2max_ each and also ran outside for 30 minutes at their preferred speed) while measuring their energy expenditures using SenseWear Armband and indirect calorimetry. Yet another example for divergent data on SenseWear Armband measurement precision was reported by Gastin et al. (20). In their study, 26 active adults completed a well-defined 90-minute activity session consisting of walking, jogging, running, or a sport-simulated circuit. Probands wore 2 accelerometers—the SenseWear Armband and the ActiGraph GT3X—and a portable gas analyzer that was used as a criterion measure. As a final result, it was concluded that the SenseWear Armband overestimated energy expenditures at lower intensities, whereas underestimating energy expenditures at higher intensities. Taken together, the results of these validation studies highlight the difficulties in precise energy measurements for the different modes of physical activity. But when energy expenditure is integrated over longer time periods, the measurement error becomes less pronounced, and overall estimations tend to be more accurate. Our findings indicate that the total number of steps per day and the amount of vigorous activity showed a positive trend toward patients in remission, although this did not reach statistical significance. Nevertheless, PAL levels were significantly higher in remission vs active disease. The PAL measured by the SenseWear BodyMedia Armband integrates information from a biaxial accelerometer and other physiological sensors (e.g., heat flux, temperature, and galvanic skin response) to provide estimates of energy expenditure using a proprietary algorithm. The number of steps per day and the amount of vigorous activity per day do not exclusively cover the value of the PAL, and therefore, these results are not contradictory. Overall, we believe that the SenseWear Armband is an easily accessible tool to objectively measure habitual energy expenditure with reasonable accuracy, and therefore, we used it in the current study. Moreover, the SenseWear Armband is well suited to capture other functions such as sleep efficiency.

Patient-reported outcomes—including disease-specific quality of life—are becoming an increasing priority in the management of patients with IBD, which is reflected by current initiatives within the FDA Clinical Outcome Assessment Qualification Program (https://www.fda.gov/Drugs/default.htm). A positive impact of physical activity on quality of life has been demonstrated for a multitude of chronic conditions, such as type II diabetes (21). In patients with IBD, moderate-to-vigorous physical activity has been shown to improve patients' reported quality of life (22). In addition, a positive effect of physical activity during exercise programs on IBD patients' quality of life has been reported (23,24). However, many studies did not objectively measure habitual physical activity in patients with IBD but relied on questionnaires (25). There is evidence that subjectively measured PALs may overestimate the objectively measured PALs, so to fully investigate the complexity of physical activity, a parallel use of subjective and objective instruments is recommended (26). In our study, subjectively measured PALs evaluated by the IPAQ did not significantly overestimate PALs when compared with objectively measured PALs by accelerometry.

It should be conceded, however, that many of the published studies focused on patients in disease remission, and that not all patients are open to exercise programs. In this study, one goal was therefore to objectively measure habitual physical activity in patients with IBD during their general life through a wearable accelerometer—the SenseWear Armband. In a cohort of 39 pediatric IBD patients, Werkstetter et al. (12) investigated habitual physical activity using the SenseWear Pro2 Armband and HRQoL compared with healthy controls. The authors could not find a significant correlation between HRQoL and habitual physical activity. Potential reasons might be the relatively small sample size and the fact that only patients in remission or with only mild disease activity were included. Consistent with our findings, the study reported a trend toward lower habitual PALs in pediatric IBD patients compared with healthy age- and sex-matched controls. Because we found significantly higher PAL levels in remission vs active disease, we further performed subanalyses on PAL levels in patients with UC and patients with CD. Notably, we could not find a significant difference between PAL levels between the 2 IBD entities (see Figure, Supplementary Digital Content 1, http://links.lww.com/CTG/A117). Moreover, we did not see a difference in the impact of disease activity on PAL levels between patients with CD and patients with UC, and in addition, there was no difference in PAL's effect on quality of life between patients with UC and patients with CD. These results underline the important global impact of PAL on IBD patients' quality of life independent of the IBD entity. In our study, both groups—those in active disease and those in remission—did not reach 10.000 steps per day in median as recommended by the WHO (25), which may indicate that patients with IBD tend to have a lower habitual physical activity in general. This is consistent with a recent study by van Langenberg et al., which measured habitual physical activity in 49 patients with CD through a triaxial accelerometer (GT3X) for 7 subsequent days in comparison with 30 matched healthy controls. The authors could show that patients with CD performed less total accelerometer counts and completed fewer bouts of moderate-vigorous intensity exercise than the healthy controls did (27). As measured by the accelerometer, sleep efficiency was lower in patients with CD than in healthy controls. Our study expands on these results by showing that sleep efficiency is dependent on the activity of IBD.

Vitamin D metabolism seems to play a pivotal role in the pathogenesis and clinical course of many immunologic diseases such as multiple sclerosis (28), systemic lupus erythematodes (29), and IBD (30). Many studies tried to elucidate the interrelation between vitamin D status and IBD activity status with somewhat conflicting results but generally lower vitamin D levels in active IBD (31). With respect to quality of life, a positive correlation has been reported for irritable bowel syndrome (32), but this has not been studied previously in IBD. Our data did not reveal significant differences in vitamin D levels between groups (active disease and remission), and we also did not find an association between vitamin D levels and HRQoL. One explanation might be that vitamin D levels are closely measured in our outpatient clinic, and deficiencies are thus closely supplemented.

Our study has several limitations, primarily arising from the heterogenous patient population. When we compared calprotectin levels between the 2 groups, we could not find a significant difference between the groups, although there was a clear trend toward higher calprotectin levels within the group in active IBD. This might be due to the fact that there were some patients with elevated calprotectin levels within the remission group. Unfortunately, we do not have underlying endoscopic diagnostic findings for these patients, so we cannot rule out that there was endoscopic disease activity within some patients of the remission group. But by defining distinct groups using valid clinical scoring systems—“remission” defined as a HBI of <5 (14) or a SCCAI of <4 (15) or “active disease” (HBI > 7 or SCCAI > 6)—we chose strict cutoff values to define clearly separate groups. Furthermore, one should keep in mind that fecal calprotectin is a variable parameter, which is not only impacted by intraluminal IBD activity but also by other environmental factors such as proton-pump inhibition, gastrointestinal infections, or other gastrointestinal diseases such as reflux esophagitis (33,34). Moreover, there is evidence these days about a certain interindividual inconsistency of calprotectin levels between patients with IBD. So, it happens that when groups of patients with IBD and different levels of disease activity are compared, considerable overlaps in calprotectin concentrations between the groups are found (35,36).

Further on, we noted marked differences in baseline physical activity status and skeletal muscle mass, which may particularly influence the results of our cross-sectional approach. Orthopedic diseases and other diseases limiting habitual exercise levels (e.g., heart insufficiency) were counted as exclusion criteria for this study, but nevertheless intrinsic habitual activity levels may differ between individuals, and this might deform the results. One possible confounding factor for habitual PALs is working status, for which we did not find a significant difference in active disease vs remission. When comparing PAL levels between the 2 groups, it was taken into account whether the participants were full-time or part-time employed and whether they were unemployed. There was no significant difference in the working status between the groups. Further occupational classifications were not evaluated. Because habitual PALs were objectively measured by accelerometry, the impact of the individual's profession on the activity levels should not bias our results since nonetheless overall daily activity levels were measured on a 24-hour basis for 7 ongoing days.

In our study, we could not find a significant difference in BMI between the groups and IBD entities. This might be due to the fact that we included patients with moderate-highly active IBD during an early stage of their IBD flare to rule out that PALs were biased by the initiation of rapid-acting anti-inflammatory therapies (e.g., prednisolone). Therefore, the patients were included at an early time point of IBD activity onset, and this might have led to the equal BMI distribution between the groups.

Taken together, our study is—to the best of our knowledge—the first to reveal a close association between objectively measured habitual PALs, HRQoL, and disease activity in patients with IBD. Within the framework of patient-reported outcomes, this points to an important function of habitual physical activity as a coeffector, which can be easily assessed using wearables as modulator of patients' quality of life.

## CONFLICTS OF INTEREST

**Guarantor of the article:** Miriam Wiestler, MD.

**Specific author contributions:** Miriam Wiestler and Fabian Kockelmann share the first authorship. Masoumeh Attaran-Bandarabadi and Oliver Bachmann share the last authorship. M.W., M.A.B., U.T., and O.B. conceived and designed the study. F.K., M.W., M.K., and M.A.B. acquired the data. F.K., M.W., M.P.M., and O.B. analyzed and interpreted the data. M.W., F.K., and O.B. drafted the article; all authors revised it critically for important intellectual content, approved the version to be published, and agreed to be accountable for all aspects of the work, thereby ensuring that questions related to the accuracy or integrity of any part of the work are appropriately investigated and resolved. This report includes work performed by F.K. in fulfillment of the requirements for his doctoral thesis.

**Financial support:** O.B. reports grants and personal fees from Takeda Pharma, personal fees from Shield Therapeutics, grants and personal fees from Ferring Pharmaceuticals, grants and personal fees from Pfizer, personal fees from CED Service GmbH, grants and personal fees from Novartis AG, personal fees and other from German Society for Digestive and Metabolic Diseases, grants and personal fees from Janssen Pharmaceutica, grants and personal fees from Merck Sharp & Dohme, grants from the German Center for Infection Research, grants and personal fees from Biogen, grants and personal fees from AbbVie, personal fees from Astellas Pharma, grants and personal fees from Falk Pharma GmbH, grants and personal fees from Bristol-Myers Squibb, and personal fees from Immundiagnostik, outside the submitted work. For the remaining authors, none were declared.

**Potential competing interests:** None to report.

**Study Highlights**

**WHAT IS KNOWN**

✓ Patients with IBD suffer from reduced disease-related quality of life.

**WHAT IS NEW HERE**

✓ We objectively measured the association between habitual physical activity, IBD activity, and IBD-related quality of life.

**TRANSLATIONAL IMPACT**

✓ Physical activity can be interpreted as a substantial comodulator to improve IBD-related quality of life in patients with IBD.

## Supplementary Material

SUPPLEMENTARY MATERIAL
