# Supplementary figures and images for: Quality of Life Is Associated With Wearable-Based Physical Activity in Patients With Inflammatory Bowel Disease: A Prospective, Observational Study
Source: Clin Transl Gastroenterol. 2019 Nov 1;10(11):e00094. doi: 10.14309/ctg.0000000000000094 (PMC6890277; doi:10.14309/ctg.0000000000000094)

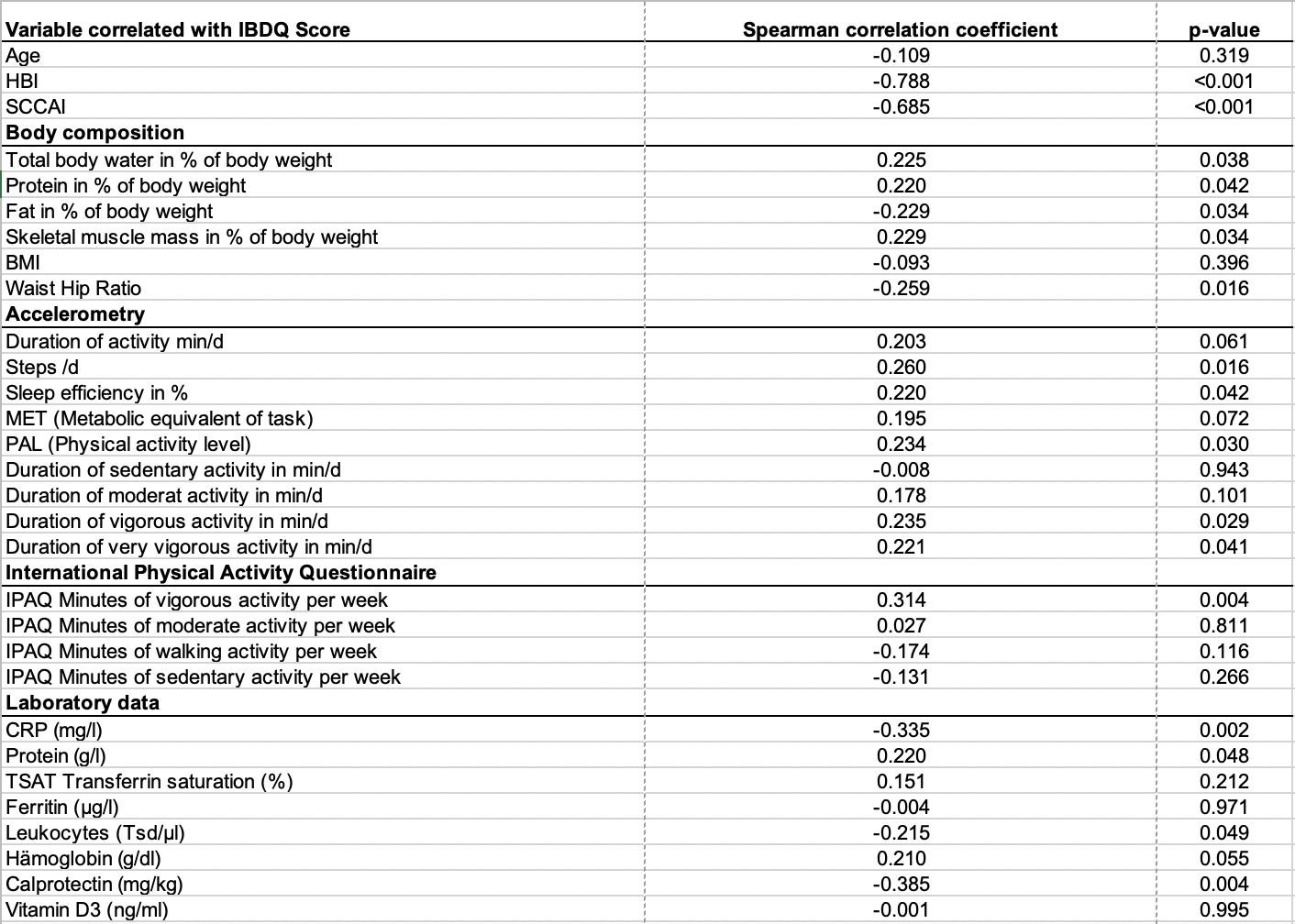

Supplement: SUPPLEMENTARY MATERIAL [file ct9-10-e00094-s001.tif]

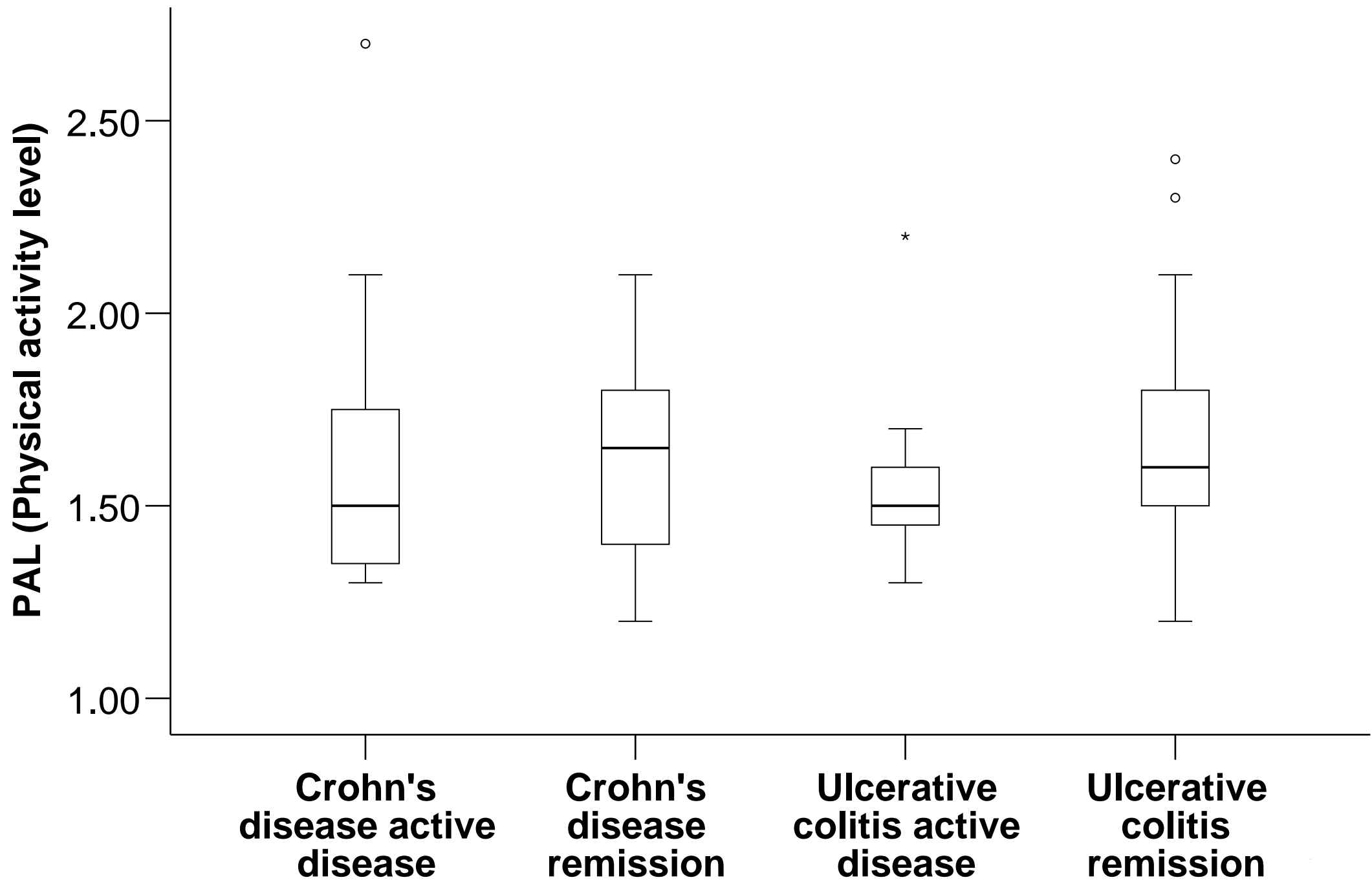

Supplement: SUPPLEMENTARY MATERIAL [file ct9-10-e00094-s002.pdf]
